# Robotic *versus* open pancreatoduodenectomy in patients with obesity: propensity score-matched multicentre study

**DOI:** 10.1093/bjsopen/zrag107

**Published:** 2026-10-27

**Authors:** Valentina Valle, Tiziana Marchese, Benedetto Ielpo, Annalisa Comandatore, Marcello Spampinato, Leonardo Borgioli, Gregorio Di Franco, Pier Cristoforo Giulianotti, Luca Morelli, the PANFRAIL Collaborative Group, M De Pastena, A Esposito, R Salvia, G Ietto, R Delpini, T Hackert, F Gehrisch, K Afshar-Bakshloo, F G Uzunoglu, S Ross, G Vanterpool, F Berrevoet, L Standaert, D Citterio, V Mazzaferro, M Cereda, T Ishizawa, A Malpaga, M Ciola, F Gasparin, F Maroso, R Romito, E Poletto, A Ruzzenente, N Furbetta, S Guadagni, G Marchegiani, F Cobellis, A Iacomino, G Zanus, F Giannone, F Matarazzo, M Scheiterle, R Rhaiem, P Zimmermann, J Martinie, R Memba Ikuga, M Defez, E Jovine, L Mastrangelo, E Vicente, V Ferri, Y Quijano, R Caruso, S Delis, C J Wray, F Maffeis, T Perra, A Porcu, A Anselmo, L E Conte, M C Giglio, R I Troisi, M P F Dorma, E Rosso, A Fassari, G Barbato, A Belli, R Memeo, R Filippo, G Garulli, M Giordano, N Machairas, M Masetti, M F Offi, G Sergeant, Y Depaifve, B K P Goh, Y X Koh, Y Masuda

**Affiliations:** Division of General, Minimally Invasive, & Robotic Surgery, University of Illinois at Chicago, Illinois, USA; General and HPB Surgery Department, Vito Fazzi Hospital, Lecce, Italy; HPB Unit, Hospital Del Mar, Pompeu Fabra University, Barcelona, Spain; General Surgery Unit, Department of Translational Research and New Technologies in Medicine and Surgery, University of Pisa, Pisa, Italy; General and HPB Surgery Department, Vito Fazzi Hospital, Lecce, Italy; Department of Electrical and Computer Engineering, College of Engineering, University of Illinois at Chicago, Chicago, Illinois, USA; General Surgery Unit, Department of Translational Research and New Technologies in Medicine and Surgery, University of Pisa, Pisa, Italy; Division of General, Minimally Invasive, & Robotic Surgery, University of Illinois at Chicago, Illinois, USA; General Surgery Unit, Department of Translational Research and New Technologies in Medicine and Surgery, University of Pisa, Pisa, Italy

**Keywords:** robotic surgery, minimally invasive surgery, propensity score matching, postoperative pancreatic fistula, surgical outcomes

## Abstract

**Background:**

Obesity increases morbidity after major abdominal surgery. Although the use of robotic pancreatoduodenectomy (RPD) is increasing, evidence of its outcomes in patients with obesity remains limited. This study compared perioperative outcomes of RPD *versus* open pancreatoduodenectomy (OPD) in patients with obesity.

**Methods:**

This was a retrospective multicentre cohort study of patients with a body mass index (BMI) ≥ 30 kg/m^2^ undergoing RPD or OPD between 2012 and 2024. Propensity score matching (PSM) was used to minimize selection bias. The primary outcome was severe complications (Clavien–Dindo ≥ IIIa). Secondary outcomes included blood loss, clinically relevant postoperative pancreatic fistula (CR-POPF), clinically relevant delayed gastric emptying (CR-DGE), wound infection, length of hospital stay (LOS), 90-day mortality, and R0 resection. Factors independently associated with severe complications and CR-POPF were investigated using multivariable logistic regression. Fistula risk was classified based on the Updated Alternate Fistula Risk Score (Pancreatic Fistula Risk Score, PFRS).

**Results:**

In all, 591 patients from 31 centres were analysed. After PSM, 166 patients undergoing RPD were compared to 166 undergoing OPD. In the PSM cohort, RPD was associated with significantly lower rates of severe complications (15.7% *versus* 27.7%; *P* = 0.007), CR-POPF (8.4% *versus* 18%; *P* = 0.011), CR-DGE (9% *versus* 18.7%; *P* = 0.022), and wound infection (4.2% *versus* 21%; *P* < 0.001). Median blood loss was lower in the RPD than OPD group (100 *versus* 200 ml; *P* < 0.001), as was median LOS (11 *versus* 17 days; *P* < 0.001). No differences were observed in 90-day mortality or R0 resection rates. Charlson co-morbidity index (odds ratio (OR) 0.68, 95% confidence interval (CI) 0.59-0.83, *P* = 0.003), robotic approach (OR 0.39, 95%CI 0.18-0.60, *P* = 0.002), and institutional experience of ≥ 20 robotic resections (OR 0.36, 95%CI 0.27-0.52, *P* = 0.007) were independently associated with lower CR-POPF risk, whereas a higher PFRS was associated with increased CR-POPF risk (OR 1.73, 95%CI 1.02-1.85, *P* = 0.032). Similarly, institutional experience of ≥ 20 robotic resections was independently associated with a lower incidence of severe complications (OR 0.39, 95%CI 0.22-0.81, *P* = 0.003), whereas a higher PFRS independently predicted severe complications (OR 1.93, 95%CI 1.07-2.31, *P* = 0.006).

**Conclusions:**

RPD is a safe and effective alternative to OPD in patients with obesity when performed in experienced centres. These findings support the adoption of RPD in select high-BMI populations in experienced institutions.

## Teaser

This multicentre propensity score-matched study evaluated outcomes of robotic *versus* open pancreatoduodenectomy in 591 patients with obese across 37 international centres. Robotic surgery was associated with significantly lower postoperative morbidity, including reduced clinically relevant postoperative pancreatic fistula, severe complications, and wound infections. These findings highlight the value of robotic approaches in this understudied high-risk population and underscore the importance of institutional experience in optimizing outcomes.

## Introduction

Obesity affects an estimated 2.11 billion adults and 493 million young people worldwide, representing a major public health challenge. By 2050, it is projected that more than half of all adults and nearly one-third of children and adolescents will be overweight or obese^1^.

The growing prevalence of obesity poses significant challenges for healthcare systems, particularly in the setting of complex surgery. Obesity, defined as a body mass index (BMI) ≥ 30 kg/m^2^, has been associated with increased postoperative morbidity and mortality^1^. These risks are especially relevant in patients undergoing major surgical procedures such as pancreatoduodenectomy (PD).

PD is a technically demanding operation performed for malignancies of the pancreas, duodenum, or periampullary region. Although mortality after PD has fallen to below 3% in high-volume centres, reflecting advances in surgical techniques and perioperative care, morbidity remains substantial, ranging from 27% to 43%^2^. Common complications include surgical site infections, delayed gastric emptying (DGE), and clinically relevant postoperative pancreatic fistula (CR-POPF). These complications are often more pronounced in patients with obese, whose elevated BMI can impair surgical exposure, increase technical difficulty, and prolong recovery^3^.

Over the past decade, minimally invasive pancreatic surgery has gained increasing acceptance, with robotic platforms facilitating the adoption of robotic pancreatoduodenectomy (RPD)^4,5^. Compared with open pancreatoduodenectomy (OPD), RPD may offer advantages in terms of precision, reduced surgical trauma and lower wound-related morbidity, as well as shorter length of hospital stay (LOS), while maintaining oncological adequacy in appropriately selected patients and experienced centres^6–9^.

Although RPD has been demonstrated to be as safe and effective as OPD in non-obese patients, its role in obese individuals remains poorly characterized^10^. Obesity introduces distinct technical and physiological challenges that may influence the feasibility and safety of RPD, underscoring the need for focused evaluation in this subgroup^11^.

The aim of the present study was to compare intraoperative and postoperative outcomes of RPD *versus* OPD in patients with a BMI ≥ 30 kg/m^2^.

## Methods

### Study design and participants

This multicentre international observational cohort study included consecutive patients with BMI ≥ 30 kg/m^2^ who underwent PD by either an open or robotic approach between January 2012 and December 2024. Contributors were organized under the Pancreatic Resections in Frail Patients (PANFRAIL) Collaborative Group, which focuses on outcomes of pancreatectomy in frail populations, including elderly or obese patients. Although the PANFRAIL collaborative network includes 37 participating centres, only the 31 institutions contributing eligible patients with obesity undergoing PD were included in the present study. To ensure procedural standardization and surgeon proficiency, participating centres were required to include only procedures performed by surgeons with a minimum experience of at least 50 OPDs. For centres performing RPD, a ≥ 10-procedure requirement was used as an inclusion threshold to ensure minimal exposure and data reliability, rather than to define procedural proficiency. Only robotic procedures performed with the da Vinci Surgical System were included. Patients undergoing vascular resections or multivisceral resections, as well as those with locally advanced disease, were excluded to improve intercentre comparability and to reduce confounding related to case complexity.

Given the multicentre study design, surgical and postoperative management protocols followed local standards. The included institutions had distinct perioperative protocols, experience, and procedural profiles (open-only, robotic-only, or mixed programmes). The choice of surgical approach was determined by surgeon preference, patient characteristics, and institutional resources.

The coordinating centre compiled a pseudo-anonymized master database from institutional data sets, each coded by its respective centre and containing predefined variables of interest. Data management and analysis were conducted in accordance with the principles of the Declaration of Helsinki^12^. Ethics approval was obtained at the coordinating centre (Hospital del Mar Research Institute; Reference 2024/11354/I). This study complies with the Strengthening the Reporting of Observational Studies in Epidemiology (STROBE) guidelines^13^ and Revised Strengthening the Reporting of Cohort, cross-sectional and case-control studies in surgery (STROCSS) guideline^14^. The STROBE checklist is provided in *Appendix S1*.

The primary outcome was severe postoperative complications (Clavien–Dindo ≥ IIIa)^15^. Secondary outcomes included conversion to open surgery, operative time, intraoperative blood loss, Comprehensive Complication Index (CCI) score, CR-POPF, clinically relevant DGE (CR-DGE), clinically relevant post-pancreatectomy haemorrhage (CR-PPH), bile leakage, wound infection, blood transfusion requirement, reoperation, LOS, readmission, 90-day mortality, and R0 resection.

### Data collection and definitions

Preoperative data included patient demographics, tumour characteristics, intraoperative findings, and histopathological results. Demographic variables were age, sex, BMI, American Society of Anesthesiologists (ASA) grade, co-morbidities (cardiovascular disease, respiratory disease, chronic kidney disease, and diabetes) and the Charlson co-morbidity index^15^. Patients were categorized into two groups: RPD and OPD. All patients in the RPD group were analysed according to the intention-to-treat principle. Procedures requiring intraoperative conversion to open surgery were retained within the robotic group for all analyses. No hybrid procedures were included and procedures classified as RPD were all performed as fully robotic operations.

Surgeon-level robotic volumes and cumulative operative experience were not uniformly available across participating sites; therefore, robotic expertise was assessed at the institutional level. In particular, institutional experience was dichotomized as < 20 *versus* ≥ 20 previous RPDs based on published learning-curve data^16^.

Intraoperative variables included conversion to open surgery, operative time, estimated blood loss, pancreatic texture, and the pancreatic Fistula Risk Score (PFRS). The PFRS was calculated intraoperatively using the updated alternative Fistula Risk Score, which incorporates four variables: sex, BMI, pancreatic duct diameter, and gland texture^17,18^. Postoperative complications occurring within 90 days were classified according to the Clavien–Dindo system^15^ and summarized using the CCI^19^. Severe complications were defined as Clavien–Dindo grade ≥ IIIa^20^. POPF, DGE and PPH were defined and graded according to the International Study Group of Pancreatic Surgery criteria, with clinically relevant events including grades B and C^20,21^.

Biliary leakage was defined according to the International Study Group of Liver Surgery criteria^22^ as a bilirubin concentration in the drain fluid at least three times the serum bilirubin level on or after postoperative day 3.

LOS was calculated as the number of days from surgery to hospital discharge.

Histopathological evaluation included tumour size and resection margins, assessed by experienced pathologists according to local or national protocols. R0 resection was defined as the absence of microscopic residual disease at any surgical margin^23^.

### Statistical analysis

Data were analysed using R software, version 4.2.2 (R Foundation for Statistical Computing, Vienna, Austria). Normally distributed variables are expressed as the mean with standard deviation (s.d.), whereas variables with a skewed distribution are presented as the median with interquartile range (i.q.r.). Categorical variables are summarized as frequencies and percentages and were compared using the χ^2^ test. Continuous variables were compared using Student’s *t* test or the Mann–Whitney *U* test, as appropriate. Statistical significance was defined as *P* < 0.05, two-tailed. All effect estimates are reported with their 95% confidence interval (c.i.). Separate logistic regression models were constructed to identify factors associated with CR-POPF and severe postoperative complications in the overall cohort. Candidate variables were selected *a priori* based on clinical relevance and previous literature and were first explored in univariable analyses. Variables with *P* < 0.05 in univariable analysis were entered into the multivariable models. To reduce redundancy and collinearity, variables reflecting overlapping constructs were not simultaneously forced into the same model. In particular, ASA grade was not retained together with the Charlson co-morbidity index because both reflect baseline clinical status and the Charlson co-morbidity index was preferred as a more granular measure of co-morbidity burden. BMI was also not retained in the final multivariable models because all included patients were obese (BMI ≥ 30 kg/m^2^), which limited its discriminatory value within the study population. Results are reported as odds ratios (ORs) with their 95% c.i. Complete-case analysis was applied.

### Propensity score matching

To improve comparability between groups and reduce selection bias, propensity score matching (PSM) was performed. RPD was set as the reference group for comparison with open surgery^24–26^. Propensity scores were constructed using logistic regression. Covariates were selected *a priori* based on clinical relevance and previous evidence identifying factors associated with treatment allocation and perioperative outcomes in pancreatic surgery; only variables available before surgery were considered. Included covariates were sex, age, BMI, ASA grade (I–II *versus* III–IV), Charlson co-morbidity index ≥ 1, benign *versus* malignant diagnosis, previous abdominal surgery, tumour size, and institutional robotic experience (≥ 20 previous RPDs). Because the proportion of patients who received neoadjuvant treatment was low (56 patients), this variable was not included in the propensity score model. A 1 : 1 greedy matching algorithm was applied with a calliper of 0.25 standard deviations. Covariate balance before and after PSM was assessed using absolute standardized mean differences, with values < 0.1 considered indicative of adequate balance. Missing data were handled using a complete-case approach. Patients with missing information in key baseline covariates or outcome variables were excluded, and no imputation procedures were performed. The extent of missingness for baseline variables was assessed before matching, and the final cohort analysed included only patients with complete data for variables required for propensity score estimation and outcome analysis^13^.

In the matched cohort, categorical variables were compared using McNemar’s χ^2^ test, whereas continuous variables were compared using the Wilcoxon signed-rank test^27^.

PSM was performed at the patient level and did not account for clustering by individual centres.

## Results

### Participants

In all, 699 patients with a BMI ≥ 30 kg/m^2^ who underwent PD between 2012 and 2024 were identified across 31 centres. Of these patients, 591 met the inclusion criteria and were included in the analysis: 407 (68.9%) in the OPD group and 184 (31.1%) in the RPD group (*Fig. 1*). The median number of enrolled patients per participating centre was 10 (i.q.r. 3–24). The patient contribution of each centre is reported in *Table S1*. After 1 : 1 PSM, two well balanced cohorts were generated, with 166 patients in each of the RPD and OPD groups.

**Figure 1:**
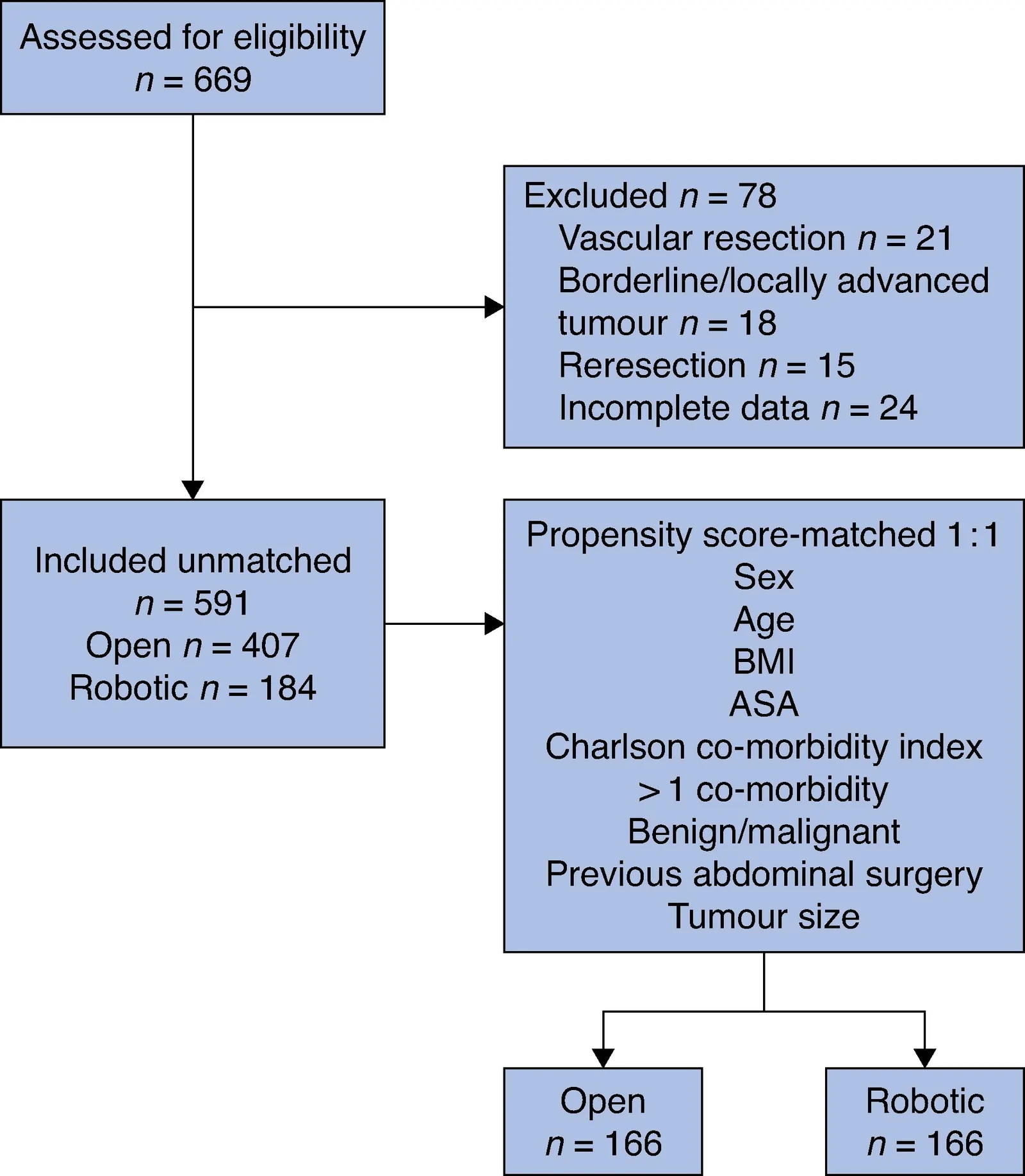
Diagram illustrating patient selection, inclusion, and exclusion criteria for the study cohorts BMI, body mass index; ASA, American Society of Anesthesiologists.

### Patient characteristics

Baseline characteristics before and after PSM are presented in *Table 1*. In the unmatched cohort (591 patients), 407 patients (68.9%) underwent OPD and 184 (31.1%) underwent RPD.

**Table 1:** Baseline characteristics of patients undergoing RPD and OPD before and after 1 : 1 propensity score matching

	Before PSM (overall *n* = 591)				After PSM (overall *n* = 332)			
	OPD (*n* = 407)	RPD (*n* = 184)	SMD	*P*	OPD (*n* = 166)	RPD (*n* = 166)	SMD	*P*
**Sex**								
Male	238 (58.5%)	80 (43.5%)	0.12	0.101	78 (47%)	75 (45%)	0.092	0.32
Female	169 (41.5%)	104 (56.5%)			88 (53%)	91 (55%)		
Age at surgery (years), median (i.q.r.)	68 (51–70)	66 (52–71)	0.177	0.77	66 (51–70)	66 (52–70)	0.081	0.78
BMI (kg/m^2^), mean(s.d.)	32.81(0.79)	32.72(0.39)	0.06	0.735	32.78(0.81)	32.69(0.81)	0.061	0.453
**ASA grade**			0.14	< 0.001			0.08	0.622
I–II	115 (28.3%)	92 (50%)			83 (50%)	79 (47.5%)		
III–IV	292 (71.7%)	92 (50%)			83 (50%)	88 (52.5%)		
Charlson co-morbidity index, mean(s.d.)	5.8(1.39)	6.1(1.23)	0.09	0.31	5.9(1.21)	6(1.15)	0.071	0.45
> 1 co-morbidity	191 (46.9%)	75 (40.7%)	0.17	0.041	81 (48.8%)	83 (50%)	0.086	0.76
**Medical history**								
Cardiovascular disease	180 (44%)	56 (30.4%)		0.27	61 (36.7%)	49 (29.5%)		0.16
Pulmonary disease	27 (6.6%)	17 (9.2%)		0.9	10 (15.5%)	13 (7.8%)		0.23
Kidney disease	29 (7.1%)	6 (3.2%)		0.62	6 (3.6%)	6 (3.6%)		1
Diabetes	73 (17.9%)	25 (13.5%)		0.74	41 (24.7%)	22 (13.2%)		0.24
Malignant tumour	225 (55.3%)	53 (23.8%)	0.19	0.047	45 (27%)	49 (29.5%)	0.681	0.073
PDAC	146	29			25	28		
Cholangiocarcinoma	36	11			13	10		
Ampullary carcinoma	25	6			5	5		
NEC	18	7			2	6		
Previous abdominal surgery	71 (17.4%)	22 (12%)	0.12	0.21	21 (12.6%)	21 (12.6%)	0.096	0.791
Tumour size (cm), mean(s.d.)	2.8(1.1)	2.3(1.3)	0.05	0.173	2.5(1)	2.3(1.2)	0.043	0.643
Preoperative biliary stenting	45 (11%)	13 (7.1%)	0.27	0.127	13 (7.8%)	12 (7.2%)	0.32	0.218

Values are *n* (%) unless otherwise stated. RPD, robotic pancreatoduodenectomy; OPD, open pancreatoduodenectomy; PSM, propensity score matching; SMD, standardized mean difference; i.q.r., interquartile range; BMI, body mass index; s.d., standard deviation; ASA, American Society of Anesthesiologists; PDAC, pancreatic ductal adenocarcinoma; NEC, neuroendocrine carcinoma.

Before matching, patients in the OPD group had a significantly higher proportion of patients with ASA grade III–IV (71.7% *versus* 50%; *P* < 0.001) and a greater likelihood of having > 1 co-morbidity (46.9% *versus* 40.7%; *P* = 0.041). A higher proportion of patients in the OPD than RPD group underwent surgery for malignant tumours (55.3% *versus* 23.8%; *P* = 0.047). No significant differences were observed between the RPD and OPD groups in terms of sex, age, BMI, Charlson co-morbidity index, history of previous abdominal surgery, tumour size, or rate of preoperative biliary stenting. After PSM, the RPD and OPD groups (166 patients in each group) were well balanced, with no statistically significant differences across baseline demographic or clinical variables, and covariate balance was adequate, with all standardized mean differences below 0.1 (*Table 1*).

### Intraoperative and postoperative outcomes

Outcomes for the overall and the propensity score-matched cohorts are summarized in *Table 2*. After PSM, median operative time did not differ significantly between the OPD and RPD groups (390 *versus* 430 min, respectively; *P* = 0.076); however, the RPD group experienced significantly lower median intraoperative blood loss (100 *versus* 200 ml; *P* < 0.001) and had fewer patients who underwent intraoperative blood transfusions (12% *versus* 18.7%; *P* = 0.03). The conversion rate from robotic to open surgery was 12.5% overall (23 patients) and 11.4% (19) after matching. After surgery, the robotic approach was associated with lower rates of complications, including CR-POPF (8.4% *versus* 18%; *P* = 0.011), CR-DGE (9% *versus* 18.7%; *P* = 0.022), and severe complications (15.7% *versus* 27.7%; *P* = 0.007), with a substantially lower incidence of wound infections (4.2% *versus* 21.0%; *P* < 0.001). The median LOS was significantly shorter for RPD than OPD patients (11 *versus* 17 days; *P* < 0.001). There were no significant differences between the two groups in postoperative haemorrhage, reoperation, or 90-day mortality.

**Table 2:** Comparison of intraoperative and postoperative outcomes between RPD and OPD in the overall and propensity score-matched cohorts

	Before PSM			After PSM		
	OPD (*n* = 407)	RPD (*n* = 184)	*P*	OPD (*n* = 166)	RPD (*n* = 166)	*P*
Conversion		23 (12.5%)			19 (11.4%)	
Operative time (min), median (i.q.r.)	400 (190–480)	460 (270–520)	0.271	390 (190–450)	430 (270–520)	0.076
Intraoperative estimated blood loss (ml), median (i.q.r.)	220 (50–350)	90 (50–250)	< 0.001	200 (60–300)	100 (55–250)	< 0.001
Pancreatic texture soft	221 (54.3%)	91 (49.5%)	0.081	71 (42.7%)	73(44.0%)	0.63
PFRS, median (i.q.r.)	6 (0–9)	5 (0–9)	0.503	6 (0–9)	6 (0–9)	0.661
Severe complications (CD ≥ III)	88 (21.6%)	31 (16.8%)	< 0.001	46 (27.7%)	26 (15.7%)	0.007
CCI score, median (i.q.r.)	22 (12.2–100)	17.2 (12.2–100)	0.047	20.2 (12.2–100)	16.8 (12.2–100)	0.164
CR-POPF	133 (32.0%)	19 (10.3%)	< 0.001	30 (18%)	14 (8.4%)	0.011
CR-DGE	54 (13.3%)	16 (8.7%)	0.26	31 (18.7%)	15 (9.0%)	0.022
CR-PPH	24 (5.9%)	6 (3.2%)	0.14	8 (4.9%)	2 (1.2%)	0.106
Bile leakage	5 (1.2%)	2 (1%)	0.419	3 (1.8%)	2 (1.2%)	0.521
Wound infection	96 (23.6%)	9 (4.9%)	< 0.001	35 (21%)	7 (4.2%)	< 0.001
Blood transfusions	87 (21.3%)	24 (13%)	< 0.001	31 (18.7%)	20 (12%)	0.03
Reoperation	46 (11.3%)	12 (6.5%)	0.41	6 (3.6%)	5 (3%)	0.25
LOS (days), median (i.q.r.)	17 (7–31)	12 (6–28)	< 0.001	17 (7–29)	11 (6–26)	< 0.001
Readmission	12 (3.3%)	9 (5%)	0.87	8 (4.8%)	6 (3.6%)	0.286
90-day mortality	16 (4.0%)	14 (7.6%)	0.281	8 (4.8%)	11 (6.6%)	0.121
R0 status	337 (82.8%)	168 (91.3%)	0.041	142 (85.5%)	150 (90.3%)	0.23
Adjuvant treatment†	141 (64.7%)	31(63.3%)	0.723	29 (70.7%)	29 (67.4%)	0.77

Values are *n* (%) unless otherwise stated. † Only for patients with adenocarcinoma: before PSM, OPD *n* = 218 and RPD *n* = 49; after PSM, OPD *n* = 41 and RPD *n* = 43. RPD, robotic pancreatoduodenectomy; OPD, open pancreatoduodenectomy; PSM, propensity score matching; min, minutes; i.q.r., interquartile range; PFRS, pancreatic Fistula Risk Score; CD, Clavien–Dindo; CCI, Comprehensive Complication Index; CR-POPF, clinically relevant postoperative pancreatic fistula; CR-DGE, clinically relevant delayed gastric empty; CR-PPH, clinically relevant post-pancreatectomy haemorrhage; LOS, length of hospital stay.

The R0 resection rate was comparable between the RPD and OPD groups (90.3% *versus* 85.5%, respectively; *P* = 0.23).

### Predictors of CR-POPF

Logistic regression analysis was performed to identify factors associated with the development of CR-POPF (*Table 3*). In univariable analysis, the robotic approach (OR 0.38, *P* = 0.001), and institutional experience exceeding 20 previous RPDs (OR0.32, *P* = 0.002) were associated with a reduced risk of CR-POPF. The Charlson co-morbidity index was inversely associated with CR-POPF risk. Conversely, a higher PFRS (OR 1.61, *P* = 0.017) and a longer operative time (OR 1.69, *P* = 0.042) were associated with increased CR-POPF risk. In multivariable analysis, the robotic approach (OR 0.39, *P* = 0.002), a lower Charlson co-morbidity index (OR 0.68, *P* = 0.003), and institutional experience ≥ 20 RPDs (OR 0.36, *P* = 0.007) remained independently associated with a reduced risk of CR-POPF. Higher PFRS (OR 1.73, *P* = 0.032) remained an independent predictor of increased risk. Operative time was no longer significant in the multivariable model.

**Table 3:** Univariable and multivariable logistic regression analyses of factors associated with clinically relevant postoperative pancreatic fistula (*n* = 591)

	Univariable		Multivariable	
	OR	*P*	OR	*P*
Female sex	1.24 (0.97, 2.13)	0.35		
Age	1.98 (0.49, 2.7)	0.34		
Charlson co-morbidity index	0.67 (0.59, 0.82)	0.009	0.68 (0.59, 0.83)	0.003
> 1 co-morbidity	1.0 (0.91, 1.23)	0.674		
Robotic approach	0.38 (0.19, 0.61)	0.001	0.39 (0.18, 0.60)	0.002
Operative time	1.69 (1.04, 1.98)	0.042	1.52 (0.71, 1.7)	0.123
Institutional experience ≥ 20 robotic resections	0.32 (0.27, 0.55)	0.002	0.36 (0.27, 0.52)	0.007
PFRS	1.61 (1.03, 1.81)	0.017	1.73 (1.02, 1.85)	0.032

Values in parentheses are 95% confidence intervals. OR, odds ratio; PFRS, pancreatic Fistula Risk Score.

### Predictors of severe complications (Clavien–Dindo grade ≥ III)

Logistic regression analysis was performed to identify predictors of severe postoperative complications (Clavien–Dindo grade ≥ III; *Table 4*). In univariable analysis, the robotic approach (OR 0.31, *P* = 0.001) and experience of ≥ 20 previous RPDs (OR 0.38, *P* = 0.002) were associated with a lower risk of severe complications, whereas a higher PFRS was associated with increased risk (OR 1.89, *P* = 0.012). In multivariable analysis, greater institutional experience with RPD (OR 0.39, *P* = 0.003) was independently associated with a reduced risk of severe complications, whereas a higher PFRS was associated with increased risk (OR 1.93, *P* = 0.006).

**Table 4:** Univariable and multivariable logistic regression analyses of factors associated with severe complications, defined as Clavien–Dindo grade ≥ III (*n* = 591)

	Univariable		Multivariable	
	OR	*P*	OR	*P*
Female sex	1.64 (0.78, 2.56)	0.85		
Age	1.01 (0.92, 2.91)	0.71		
Charlson co-morbidity index	0.97 (0.79, 1.47)	0.32		
> 1 co-morbidity	1.8 (0.81, 2.22)	0.974		
Robotic approach	0.31 (0.21, 0.81)	0.001	0.32 (0.21, 1.86)	0.30
Operative time	1.17 (0.49, 1.38)	0.53		
Institutional experience ≥ 20 robotic resections	0.38 (0.21, 0.65)	0.002	0.39 (0.22, 0.81)	0.003
PFRS	1.89 (1.03, 2.35)	0.012	1.93 (1.07, 2.31)	0.006

Values in parentheses are 95% confidence intervals. OR, odds ratio; PFRS, pancreatic Fistula Risk Score.

## Discussion

The role of RPD has gained increasing interest, particularly in high-risk populations such as obese or elderly patients, owing to the intrinsic technical complexity of pancreatic head resection and the perioperative risks associated with frailty. Obesity introduces specific challenges, including limited surgical exposure, a thickened abdominal wall, and increased visceral adiposity, which may obscure vascular landmarks, increase blood loss, impair wound healing, and prolong recovery. These anatomical and physiological factors have historically limited the adoption of minimally invasive approaches in patients with obesity. However, robotic platforms offer high-definition three-dimensional visualization, articulated instruments, and enhanced ergonomics, potentially mitigating these limitations and enabling safer surgery in complex patients^11,28–32^. Importantly, randomized evidence comparing RPD and OPD is emerging, but patients with obesity remain considerably underrepresented in these trials^6^.

The present multicentre observational study examined perioperative outcomes of RPD *versus* OPD in patients with BMI ≥ 30 kg/m^2^, providing real-world evidence specifically focused on obese patients undergoing PD. After PSM, RPD was associated with improved perioperative outcomes, including reduced severe complications, lower blood loss, and shorter LOS, while maintaining comparable R0 resection rates. Notably, the robotic approach was also independently associated with a lower risk of CR-POPF in multivariable analysis.

Early reports on minimally invasive pancreatic surgery suggested that robotic procedures are inherently longer than open operations^28–31^. In the present series, although operative times for RPD were longer than for OPD both before and after PSM, the differences did not reach statistical significance. This finding aligns with recent evidence showing that as robotic programmes mature, operative times increasingly approximate those of open surgery, in contrast to the prolonged durations reported during the early adoption of robotic techniques^9,31–33^.

Another relevant finding of this study was the relatively low conversion rate from RPD to OPD (12.5% and 11.4% before and after PSM, respectively), consistent with published series in complex populations, where reported conversion rates range from approximately 8% to 20%^30,33^. This is particularly meaningful in patients with obesity, who are often excluded from minimally invasive resections because of technical complexity. Maintaining a low conversion rate allows more patients to benefit from minimally invasive surgery without compromising safety^6,34^.

Median intraoperative blood loss was nearly halved in the robotic group, consistent with previous reports^35,36^ attributing this advantage to improved visualization and enhanced vascular control provided by the robotic systems.

The present analysis also showed lower rates of CR-POPF, CR-DGE, and wound infection for RPD compared with OPD. The reason for the lower CR-POPF rate observed in the robotic cohort is likely multifactorial. Although selection bias cannot be fully excluded, several technical characteristics of the robotic platform may contribute to improved anastomotic outcomes. Enhanced three-dimensional visualization, tremor filtration, and articulated instruments may facilitate precise dissection and pancreatojejunostomy construction, particularly in high-risk glands such as those with soft parenchyma and small duct diameter. These hypotheses are supported by previous studies reporting favourable trends in fistula rates for RPD^36^. Moreover, in both univariable and multivariable analysis, higher experience with RPD was associated with a reduced risk of CR-POPF, supporting the role of institutional programme maturity as a determinant of pancreatic-specific outcomes in RPD. Finally, the Charlson co-morbidity index showed an inverse association with CR-POPF risk; this finding should be interpreted cautiously because, rather than implying a true protective effect, it may reflect residual selection bias inherent to retrospective analyses.

The relatively high wound infection rate observed in the OPD group should be interpreted in the context of an exclusively obese cohort of patients, in whom a large laparotomy is associated with increased surgical site infection risk.

Severe complications and overall CCI scores were also significantly reduced in the RPD group, whereas CR-PPH, bile leakage, reoperation, and 90-day mortality were comparable between the two groups. The shorter LOS among RPD patients aligns with data from randomized trials^6,33^ and supports the notion that the robotic platform may mitigate the physiological and technical challenges associated with obesity, facilitating enhanced perioperative recovery^11,37–39^. However, LOS should be interpreted cautiously because it may reflect institutional pathways, social factors, and healthcare system organization.

Overall, the present findings align with previous studies reporting comparable or improved perioperative outcomes with RPD. Giglio *et al*.^29^, using inverse probability of treatment weighting, confirmed the non-inferiority of RPD compared with OPD regarding major complications and overall perioperative safety. Similarly, Gonzalez-Abos *et al*.^5^ demonstrated non-inferior outcomes of RPD in patients with obesity stratified by the PD-ROBOSCORE, supporting the applicability of robotics in this challenging population. In contrast, the EUROPA randomized trial^40^ reported higher pancreatic-specific complications after RPD. However, that trial was conducted in a single centre with a broader case mix, including more complex resections, whereas the present analysis focused specifically on patients with obesity and excluded vascular and multivisceral resections. Differences in institutional experience and programme maturity may also have contributed to the divergent findings.

Oncological adequacy remains a critical consideration in pancreatic head resection^33–36,41,42^. Importantly, R0 resection rates in the present study were comparable between the RPD and OPD groups after PSM, consistent with literature demonstrating non-inferiority of RPD^33–36,41,42^. However, because oncological outcomes were not primary endpoints of this study, no further conclusions can be drawn on this aspect.

Notably, higher RPD experience (above the threshold of 20 previous RPDs) was independently associated with a lower risk of serious morbidity and CR-POPF in the overall cohort. This finding suggests that institutional experience with the robotic technique is a critical determinant of optimal outcomes and may partly account for the variability reported across studies. It also underscores the importance of structured implementation, credentialing processes, and dedicated training pathways when establishing robotic surgery programmes, particularly in technically demanding patients with obesity.

This study has several limitations. The retrospective design carries inherent risks of unmeasured confounding, and the multicentre international nature of the study over a prolonged study period inevitably introduces heterogeneity. Although standardized definitions were applied for major outcomes, institutional and time-related variability may have influenced recovery metrics and complication reporting.

Patient-reported outcome measures, direct measures of postoperative pain, and functional recovery were not systematically available across centres. Long-term oncological variables, including survival and recurrence patterns, were also not uniformly available and were therefore not included in the primary analysis. Furthermore, neoadjuvant therapy was infrequent in the study cohort, limiting the ability to evaluate its independent effect on outcomes.

A formal cost analysis was not performed; however, the combination of shorter hospitalization, reduced morbidity, and comparable operative times suggests that RPD may be economically sustainable, consistent with a recent single-centre study^43^ demonstrating cost-effectiveness in this setting.

The exclusion of vascular resections, multivisceral resections, and locally advanced disease restricts the analysis to a more standardized PD population and may limit generalizability to the most technically demanding scenarios, where obesity may exert the greatest impact on surgical complexity. In addition, indication bias remains an inherent limitation of this retrospective study design, because the selection of the surgical approach was influenced by surgeon preference, patient characteristics, and institutional resources. Although PSM accounted for measurable preoperative confounders, relevant factors such as tumour location, vascular proximity, radiological resectability features, and surgeon-assessed technical difficulty were not uniformly available and may have contributed to residual confounding. Moreover, because matching was performed at the patient level, variability in the contribution of OPD and RPD patients across centres could not be fully controlled. Therefore, the findings of this study should be interpreted as reflecting outcomes in obese patients undergoing standard PD in experienced centres, rather than the full spectrum of complex pancreatic head resections.

Finally, surgeon-level operative volumes and cumulative career experience were not uniformly available, and institutional volume was therefore used as a proxy for robotic expertise. This was modelled using a pragmatic threshold of 20 previous RPDs to enhance clinical interpretability, while acknowledging that alternative continuous or spline-based approaches may capture more complex volume–outcome relationships. This ≥ 20-RPD threshold, well below published^16^ learning-curve benchmarks (40–80 procedures), was pragmatically chosen as an early-experience cut-off rather than a proficiency benchmark, intended to distinguish centres at different stages of robotic programme implementation. In addition, a minimum threshold of OPD experience was required for all surgeons contributing to either group to ensure a comparable baseline level of open surgical expertise and to minimize early learning-curve effects.

In conclusion, this multicentre analysis supports RPD as a safe and effective alternative to OPD in select patients with obesity. RPD was associated with lower postoperative morbidity and shorter LOS, whereas institutional robotic experience was linked to improved outcomes. These findings support centralization and structured implementation of RPD in experienced centres.

## Collaborators

Members of the PANFRAIL Collaborative Group are: M. De Pastena, A. Esposito, R. Salvia (The Pancreas Institute, University of Verona Hospital Trust, Verona, Italy); G. Ietto, R. Delpini (University of Insubria, Varese, Italy); T. Hackert, F. Gehrisch, K. Afshar-Bakshloo, F. G. Uzunoglu (University Medical Center Hamburg-Eppendorf, Hamburg, Germany); S. Ross, G. Vanterpool (AdventHealth Tampa, Digestive Health Institute, Tampa, FL, USA); F. Berrevoet, L. Standaert (Ghent University Hospital, Ghent, Belgium); D. Citterio, V. Mazzaferro (Fondazione IRCCS Istituto Nazionale Tumori, Milan, Italy); M. Cereda (Fondazione IRCCS San Gerardo Hospital, Monza, Italy); T. Ishizawa (Graduate School of Medicine, Osaka Metropolitan University, Osaka, Japan); A. Malpaga, M. Ciola, F. Gasparin (Regional Hospital of Bolzano, Bolzano, Italy); F. Maroso, R. Romito (A. O. U. Maggiore della Carità di Novara, Novara, Italy); E. Poletto, A. Ruzzenente (University of Verona, Verona, Italy); N. Furbetta, S. Guadagni (University of Pisa, Pisa, Italy); G. Marchegiani, F. Cobellis, A. Iacomino, G. Zanus (University of Padua, Padua, Italy); F. Giannone (Azienda Ospedaliero-Universitaria SS, Antonio e Biagio e Cesare Arrigo, Alessandria, Italy; Nouvel Hopital Civil, Strasbourg, France); F. Matarazzo (Azienda USL Toscana Sud Est, Grosseto, Italy); M. Scheiterle (University Hospital Careggi, Florence, Italy); R. Rhaiem, P. Zimmermann (CHU de Reims, Reims, France); J. Martinie (Carolinas Medical Center, Charlotte, NC, USA); R. Memba Ikuga, M. Defez (Joan XXIII University Hospital of Tarragona, Tarragona, Spain); E. Jovine, L. Mastrangelo (Alma Mater Studiorum University of Bologna, IRCCS AOU of Bologna, Bologna, Italy); E. Vicente, V. Ferri, Y. Quijano, R. Caruso (Sanchinarro University Hospital, HM Hospitals Faculty of Health Sciences Camilo José Cela University, Madrid, Spain); S. Delis (Konstantopoulio General Hospital, Athens, Greece); C. J. Wray (University of Texas Health Science Center at Houston, McGovern Medical School, Houston, TX, USA); F. Maffeis (St. Mary of Angels Hospital, Azienda Sanitaria Friuli Occidentale, Pordenone, Italy); T. Perra, A. Porcu (Azienda Ospedaliero-Universitaria di Sassari, Sassari, Italy); A. Anselmo, L. E. Conte (Policlinico Tor Vergata, Rome, Italy); M. C. Giglio, R. I. Troisi (Federico II University Hospital, Naples, Italy); M. P. F. Dorma (Misericordia Hospital, Azienda USL Toscana Sud Est, Grosseto, Italy; University of Modena and Reggio Emilia, Modena, Italy); E. Rosso (Centre de Chirurgie Digestive, Institut Foie et Pancréas, Pôle Santé Sud, Le Mans, France); A. Fassari (Centre Hospitalier de Luxembourg, Luxembourg City, Luxembourg); G. Barbato (Pancreatic Surgery Unit, General Surgery Department, San Giovanni di Dio Hospital, Florence, Italy); A. Belli (Istituto Nazionale Tumori IRCCS Fondazione G. Pascale, Naples, Italy); R. Memeo, R. Filippo (F. Miulli General Regional Hospital, Acquaviva delle Fonti, Bari, Italy; Department of Medicine and Surgery, LUM University, Casamassima, Bari, Italy); G. Garulli, M. Giordano (Infermi Hospital, Rimini, Italy); N. Machairas (Laiko General Hospital, School of Medicine, National and Kapodistrian University of Athens, Athens, Greece); M. Masetti, M. F. Offi (U. O. C. Chirurgia Generale, AUSL di Imola, Ospedale S. Maria della Scaletta, Imola, Italy); G. Sergeant, Y. Depaifve (Jessa Hospital, Faculty of Medicine and Health Sciences, Hasselt, Belgium); B. K. P. Goh, Y. X. Koh, Y. Masuda (Singapore General Hospital and National Cancer Centre Singapore, Singapore).

## Supplementary Material

zrag107_Supplementary_Data

## Data Availability

The datasets generated and analysed during the current study are available under the request to corresponding author and approval of all authors.
