# Supplementary material for: Robotic versus open pancreatoduodenectomy in patients with obesity: propensity score-matched multicentre study
Source: BJS Open. 2026 Oct 27;10(5):zrag107. doi: 10.1093/bjsopen/zrag107 (PMC13711665; doi:10.1093/bjsopen/zrag107)
Supplement: Supplementary file 1 [file zrag107_supplementary_data.zip › STROBE Checklist BJS.docx]

# STROBE Checklist – Cohort Study

Manuscript Title: Robotic Pancreatoduodenectomy in Obese Patients: A Propensity Score-Matched Multicenter Study of the PANFRAIL Collaborative Group

Study Design: Retrospective multicentre cohort study

Journal: British Journal of Surgery (BJS)

| Section | Item No. | STROBE Recommendation | Reported in Manuscript (Section) |
| --- | --- | --- | --- |
| Title & Abstract | 1a | Indicate study design in title or abstract | Abstract: Retrospective multicentre cohort study |
| Title & Abstract | 1b | Provide informative and balanced abstract | Structured abstract with objectives, methods (PSM), results and conclusions |
| Introduction | 2 | Explain scientific background and rationale | Introduction section |
| Introduction | 3 | State specific objectives and hypotheses | Aim clearly stated: comparison of RPD vs OPD in BMI ≥30 |
| Methods | 4 | Present key elements of study design early | Methods – Study Design subsection |
| Methods | 5 | Describe setting, locations, and relevant dates | 32 international centres; January 2012 – December 2024 |
| Methods | 6a | Give eligibility criteria and selection methods | Inclusion/exclusion criteria clearly specified |
| Methods | 6b | Describe matching criteria and method | 1:1 propensity score matching; caliper 0.25 SD; predefined covariates |
| Methods | 7 | Clearly define outcomes, exposures, predictors, confounders | CR-POPF, severe complications, LOS, blood loss, operative time |
| Methods | 8 | Data sources and measurement methods | Multicentre pseudo-anonymised database; ISGPS definitions applied |
| Methods | 9 | Describe efforts to address bias | PSM; standardized complication definitions; predefined covariates |
| Methods | 10 | Explain study size determination | 591 included patients; no formal power calculation performed |
| Methods | 11 | Explain handling of quantitative variables | Continuous variables as mean±SD or median (IQR); ASA dichotomised |
| Methods | 12a | Statistical methods to control confounding | Logistic regression; multivariable models |
| Methods | 12b | Methods for subgroup or interaction analyses | Centre experience ≥20 RPD threshold analysis |
| Methods | 12c | Handling of missing data | Complete-case analysis; no imputation performed |
| Results | 13a | Report numbers at each stage | 699 identified → 591 included → 166+166 matched |
| Results | 13b | Give reasons for exclusions | Exclusion criteria detailed in Methods and Figure 1 |
| Results | 14 | Provide descriptive data | Baseline characteristics in Table 1 |
| Results | 15 | Report outcome data | Intra- and postoperative outcomes in Table 2 |
| Results | 16 | Main results with estimates and precision | ORs with 95% CI and p-values reported |
| Results | 17 | Report other analyses performed | Predictors of CR-POPF and severe complications (Tables 3–4) |
| Discussion | 18 | Summarise key results | Opening paragraph of Discussion |
| Discussion | 19 | Discuss limitations | Dedicated limitations section |
| Discussion | 20 | Provide cautious interpretation | Contextualised within current literature |
| Discussion | 21 | Discuss generalisability | Multicentre design; applicability to experienced centres |
| Other Information | 22 | Funding and conflicts of interest | No funding; no conflicts declared |
