# Supplementary material for: Robotic versus open pancreatoduodenectomy in patients with obesity: propensity score-matched multicentre study
Source: BJS Open. 2026 Oct 27;10(5):zrag107. doi: 10.1093/bjsopen/zrag107 (PMC13711665; doi:10.1093/bjsopen/zrag107)
Supplement: Supplementary file 1 [file zrag107_supplementary_data.zip › Supplementary_Material.docx]

**Robotic versus Open Pancreatoduodenectomy in Obese Patients: A Propensity Score-Matched Multicentre Study of the PANFRAIL Collaborative Group**

Valentina Valle MD^1^*, Tiziana Marchese MD^2^*, Benedetto Ielpo MD^3^*, Annalisa Comandatore MD^4^, Marcello Spampinato MD^2^, Leonardo Borgioli^5^, Gregorio Di Franco MD, PhD^4^, Pier Cristoforo Giulianotti MD^1^**, Luca Morelli, MD, FACS, MAMSE^4^**, on behalf of the **PANFRAIL Collaborative Group**.

*These authors share the first authorship.

**These authors share the senior authorship.

**Author affiliations**

1 DIVISION OF GENERAL, MINIMALLY INVASIVE & ROBOTIC SURGERY, UNIVERSITY OF ILLINOIS AT CHICAGO, USA

2 GENERAL AND HPB SURGERY DEPARTMENT, LECCE, ITALY

3 HPB UNIT, HOSPITAL DEL MAR, POMPEU FABRA UNIVERSITY, BARCELONA, SPAIN

4 GENERAL SURGERY UNIT, DEPARTMENT OF TRANSLATIONAL RESEARCH AND NEW TECHNOLOGIES IN MEDICINE AND SURGERY, UNIVERSITY OF PISA, PISA, ITALY

5 DEPARTMENT OF ELECTRICAL AND COMPUTER ENGINEERING, COLLEGE OF ENGINEERING, UNIVERSITY OF ILLINOIS AT CHICAGO, CHICAGO, IL, UNITED STATES

**Corresponding author.**

Name: Benedetto Ielpo

Address: Passeig Maritim 25, 08034, Barcelona, Spain

E-mail: ielpo.b@gmail.com

**Supplementary Materials - Index**

| **Supplementary Appendixes** |  |
| --- | --- |
| Supplementary Appendix 1 | *page 2* |
| **Supplementary Figures and Tables** |  |
| Supplementary Table 1 | *page 6* |

**Supplementary Appendix 1.** STROBE Statement—checklist of items that should be included in reports of observational studies

|  | | **Item No.** | | **Recommendation** | **Page  No.** | | **Relevant text from manuscript** |
| --- | --- | --- | --- | --- | --- | --- | --- |
| **Title and abstract** | | 1 | | (*a*) Indicate the study’s design with a commonly used term in the title or the abstract | 5 | | Abstract: Retrospective multicentre cohort study |
|  |  |  |  | (*b*) Provide in the abstract an informative and balanced summary of what was done and what was found | 5 | | Structured abstract with objectives, methods (PSM), results and conclusions |
| **Introduction** | | | | | | |  |
| Background/rationale | | 2 | | Explain the scientific background and rationale for the investigation being reported | 7 | | Introduction section |
| Objectives | | 3 | | State specific objectives, including any prespecified hypotheses | 7 | | Aim clearly stated: comparison of RPD vs OPD in BMI ≥30 |
| **Methods** | | | | | | |  |
| Study design | | 4 | | Present key elements of study design early in the paper | 8 | | Methods – Study Design subsection |
| Setting | | 5 | | Describe the setting, locations, and relevant dates, including periods of recruitment, exposure, follow-up, and data collection | 8 | | 32 international centres; January 2012 – December 2024 |
| Participants | | 6 | | (*a*) *Cohort study*—Give the eligibility criteria, and the sources and methods of selection of participants. Describe methods of follow-up  *Case-control study*—Give the eligibility criteria, and the sources and methods of case ascertainment and control selection. Give the rationale for the choice of cases and controls  *Cross-sectional study*—Give the eligibility criteria, and the sources and methods of selection of participants | 8 | | Inclusion/exclusion criteria clearly specified |
|  |  |  |  | (*b*) *Cohort study*—For matched studies, give matching criteria and number of exposed and unexposed  *Case-control study*—For matched studies, give matching criteria and the number of controls per case | 11 | | 1:1 propensity score matching; caliper 0.25 SD; predefined covariates |
| Variables | | 7 | | Clearly define all outcomes, exposures, predictors, potential confounders, and effect modifiers. Give diagnostic criteria, if applicable | 8-10 | | CR-POPF, severe complications, LOS, blood loss, operative time |
| Data sources/ measurement | | 8* | | For each variable of interest, give sources of data and details of methods of assessment (measurement). Describe comparability of assessment methods if there is more than one group | 8-10 | | Multicentre pseudo-anonymised database; ISGPS definitions applied |
| Bias | | 9 | | Describe any efforts to address potential sources of bias | 8-10 | | PSM; standardized complication definitions; predefined covariates |
| Study size | | 10 | | Explain how the study size was arrived at | - | | 591 included patients; no formal power calculation performed |
| Quantitative variables | 11 | | Explain how quantitative variables were handled in the analyses. If applicable, describe which groupings were chosen and why | | 9-11 | Continuous variables as mean±SD or median (IQR); ASA dichotomised | |
| Statistical methods | 12 | | (*a*) Describe all statistical methods, including those used to control for confounding | | 10-11 | Multivariable logistic regression models, PSM | |
|  |  |  | (*b*) Describe any methods used to examine subgroups and interactions | | - | - | |
|  |  |  | (*c*) Explain how missing data were addressed | | 11 | Complete-case analysis; no imputation performed | |
|  |  |  | (*d*) *Cohort study*—If applicable, explain how loss to follow-up was addressed  *Case-control study*—If applicable, explain how matching of cases and controls was addressed  *Cross-sectional study*—If applicable, describe analytical methods taking account of sampling strategy | | - | - | |
|  |  |  | (*e*) Describe any sensitivity analyses | | - | - | |
| **Results** | | | | | | | |
| Participants | 13* | | (a) Report numbers of individuals at each stage of study—eg numbers potentially eligible, examined for eligibility, confirmed eligible, included in the study, completing follow-up, and analysed | | 12, Figure 1 | 699 identified → 591 included → 166+166 matched | |
|  |  |  | (b) Give reasons for non-participation at each stage | | 8; Figure 1 | Exclusion criteria detailed in Methods and Figure 1 | |
|  |  |  | (c) Consider use of a flow diagram | | Figure 1 | Fig 1 | |
| Descriptive data | 14* | | (a) Give characteristics of study participants (eg demographic, clinical, social) and information on exposures and potential confounders | | Table 1 | Baseline characteristics in Table 1 | |
|  |  |  | (b) Indicate number of participants with missing data for each variable of interest | | - | - | |
|  |  |  | (c) *Cohort study*—Summarise follow-up time (eg, average and total amount) | | 9 | 90-day | |
| Outcome data | 15* | | *Cohort study*—Report numbers of outcome events or summary measures over time | | Table 2 | Intra- and postoperative outcomes in Table 2 | |
|  |  |  | *Case-control study—*Report numbers in each exposure category, or summary measures of exposure | |  |  | |
|  |  |  | *Cross-sectional study—*Report numbers of outcome events or summary measures | |  |  | |
| Main results | 16 | | (*a*) Give unadjusted estimates and, if applicable, confounder-adjusted estimates and their precision (eg, 95% confidence interval). Make clear which confounders were adjusted for and why they were included | | 12-13, Tables | ORs with 95% CI and p-values reported | |
|  |  |  | (*b*) Report category boundaries when continuous variables were categorized | | 11 |  | |
|  |  |  | (*c*) If relevant, consider translating estimates of relative risk into absolute risk for a meaningful time period | | - | - | |

| Other analyses | 17 | Report other analyses done—eg analyses of subgroups and interactions, and sensitivity analyses | Tables 3-4 | Predictors of CR-POPF and severe complications |
| --- | --- | --- | --- | --- |
| **Discussion** | | | | |
| Key results | 18 | Summarise key results with reference to study objectives | 14 | Opening paragraph of Discussion |
| Limitations | 19 | Discuss limitations of the study, taking into account sources of potential bias or imprecision. Discuss both direction and magnitude of any potential bias | 16-17 | Dedicated limitations section |
| Interpretation | 20 | Give a cautious overall interpretation of results considering objectives, limitations, multiplicity of analyses, results from similar studies, and other relevant evidence | 14-17 | Contextualised within current literature |
| Generalisability | 21 | Discuss the generalisability (external validity) of the study results | 14-17 | Multicentre design; applicability to experienced centres |
| **Other information** | |  | | |
| Funding | 22 | Give the source of funding and the role of the funders for the present study and, if applicable, for the original study on which the present article is based | 18 | No funding; no conflicts declared |

*Give information separately for cases and controls in case-control studies and, if applicable, for exposed and unexposed groups in cohort and cross-sectional studies.

**Note:** An Explanation and Elaboration article discusses each checklist item and gives methodological background and published examples of transparent reporting. The STROBE checklist is best used in conjunction with this article (freely available on the Web sites of PLoS Medicine at http://www.plosmedicine.org/, Annals of Internal Medicine at http://www.annals.org/, and Epidemiology at http://www.epidem.com/). Information on the STROBE Initiative is available at www.strobe-statement.org.

**Supplementary Table 1.** Patient contribution of individual centers

| **Center** | **OPD (n=407)** | **RPD (n=184)** |
| --- | --- | --- |
| Hepatopancreatobiliary Surgery, AdventHealth Tampa, Digestive Health Institute, Tampa, FL, USA | 46 | 90 |
| Department of General and Pancreatic Surgery, The Pancreas Institute, University of Verona Hospital Trust, Verona, Italy | 65 | 0 |
| Department of General, Visceral and Thoracic Surgery, University Medical Center Hamburg-Eppendorf, Hamburg, Germany | 41 | 1 |
| Division Of General, Minimally Invasive, & Robotic Surgery, University Of Illinois At Chicago, Usa | 10 | 34 |
| Nouvel Hopital Civil, Strasbourg, France | 25 | 2 |
| HPB Surgery, Liver Transplantation and Pathology, Fondazione IRCCS Istituto Nazionale Tumori, Milan, Italy | 30 | 0 |
| General Surgery Unit, Department of Translational Research and New Technologies in Medicine and Surgery, University of Pisa, Pisa, Italy | 19 | 5 |
| General and Hepatobiliary Surgery, Department of Surgery, Dentistry, Gynecology and Pediatrics, University of Verona, Verona, Italy | 24 | 0 |
| General and HPB Surgery and Liver Transplantation, Ghent University Hospital, Ghent, Belgium | 23 | 0 |
| Department of Surgery, Joan XXIII University Hospital of Tarragona, Tarragona, Spain | 19 | 2 |
| Division of HPB Surgery, Department of Surgery, Carolinas Medical Center, Charlotte, NC, USA | 0 | 19 |
| Unit of General, Emergency and Transplant Surgery, ASST dei Sette Laghi, Department of Medicine and Surgery, University of Insubria, Varese, Italy | 18 | 0 |
| Department of HPB, Digestive and Endocrine Surgery, CHU de Reims, Reims, France | 16 | 0 |
| Hpb Unit, Hospital Del Mar, Pompeu Fabra University, Barcelona, Spain | 8 | 8 |
| Hepatopancreatobiliary and Liver Transplant Surgery, Department of Surgery, Oncology and Gastroenterology, DiSCOG, University of Padua, Padua, Italy | 10 | 1 |
| Department of Surgery, University of Texas Health Science Center at Houston, McGovern Medical School, Houston, TX, USA | 0 | 9 |
| Division of HPB, Minimally Invasive and Robotic Surgery, Transplantation Service Federico II University Hospital, Naples, Italy | 0 | 7 |
| Department of General Surgery, Konstantopoulio General Hospital, Athens, Greece | 5 | 0 |
| Alma Mater Studiorum University of Bologna, IRCCS AOU of Bologna, Bologna, Italy | 5 | 0 |
| General Surgery Service, Sanchinarro University Hospital, HM Hospitals Faculty of Health Sciences Camilo José Cela University, Madrid, Spain | 2 | 2 |
| Department of General and Emergency Surgery, Misericordia Hospital, Azienda USL Toscana Sud Est, Grosseto, Italy | 1 | 3 |
| General And Hpb Surgery Department, Lecce, Italy | 13 | 1 |
| Second Division of Surgery-Treviso, Department of Surgery, Oncology and Gastroenterology, DiSCOG, University of Padua, Padua, Italy | 3 | 0 |
| Division of General Surgery II and HPB Unit, A.O.U. Maggiore della Carità di Novara, Novara, Italy | 3 | 0 |
| Ospedale San Giovanni di Dio, Firenze, Italy | 3 | 0 |
| HPB and Transplant Surgery Unit, Department of Surgery, Policlinico Tor Vergata, Rome, Italy | 3 | 0 |
| Unit of Hepato-Biliary and Pancreatic Surgery, Istituto Nazionale Tumori IRCCS Fondazione G. Pascale, Naples, Italy | 3 | 0 |
| Department of Hepato-Pancreatic-Biliary Surgery, F. Miulli General Regional Hospital, Acquaviva delle Fonti, Bari, Italy; Department of Medicine and Surgery, LUM University, Casamassima, Bari, Italy | 3 | 0 |
| U.O.C. Clinica Chirurgica, Cliniche San Pietro, Azienda Ospedaliero-Universitaria di Sassari, Sassari, Italy | 3 | 0 |
| Department of Hepatobiliary-Pancreatic Surgery, Graduate School of Medicine, Osaka Metropolitan University, Osaka, Japan | 3 | 0 |
| Unit of Critical Care Surgery and Trauma-Trauma Team, University Hospital Careggi, Florence, Italy | 3 | 0 |
